# Dual transcriptional profiling of mice and *Toxoplasma gondii* during acute and chronic infection

**DOI:** 10.1186/1471-2164-15-806

**Published:** 2014-09-20

**Authors:** Kelly J Pittman, Matthew T Aliota, Laura J Knoll

**Affiliations:** Department of Medical Microbiology and Immunology, University of Wisconsin - Madison, 1550 Linden Drive, Madison, WI 53706 USA; Department of Pathobiological Sciences, University of Wisconsin-Madison, 1656 Linden Drive, Madison, WI 53706 USA

**Keywords:** *Toxoplasma*, RNA-seq, Transcriptome, Acute infection, Chronic infection, Forebrain, Metabolism, Immune response

## Abstract

**Background:**

The obligate intracellular parasite *Toxoplasma gondii* establishes a life-long chronic infection within any warm-blooded host. After ingestion of an encysted parasite, *T. gondii* disseminates throughout the body as a rapidly replicating form during acute infection. Over time and after stimulation of the host immune response, *T. gondii* differentiates into a slow growing, cyst form that is the hallmark of chronic infection. Global transcriptome analysis of both host and parasite during the establishment of chronic *T. gondii* infection has not yet been performed. Here, we conducted a dual RNA-seq analysis of *T. gondii* and its rodent host to better understand host and parasite responses during acute and chronic infection.

**Results:**

We obtained nearly one billion paired-end RNA sequences from the forebrains of uninfected, acutely and chronically infected mice, then aligned them to the genomic reference files of both *T. gondii* and *Mus musculus.* Gene ontology (GO) analysis of the 100 most highly expressed *T. gondii* genes showed less than half were shared between acute and chronic infection. The majority of the highly expressed genes common in both acute and chronic infection were involved in transcription and translation, underscoring that parasites in both stages are actively synthesizing proteins. Similarly, most of the *T. gondii* genes highly expressed during chronic infection were involved in metabolic processes, again highlighting the activity of the cyst stage at 28 days post-infection. Comparative analyses of host genes using uninfected forebrain revealed over twice as many immune regulatory genes were more abundant during chronic infection compared to acute. This demonstrates the influence of parasite development on host gene transcription as well as the influence of the host environment on parasite gene transcription.

**Conclusions:**

RNA-seq is a valuable tool to simultaneously analyze host and microbe transcriptomes. Our data shows that *T. gondii* is metabolically active and synthesizing proteins at 28 days post-infection and that a distinct subset of host genes associated with the immune response are more abundant specifically during chronic infection. These data suggest host and pathogen interplay is still present during chronic infection and provides novel *T. gondii* targets for future drug and vaccine development.

**Electronic supplementary material:**

The online version of this article (doi:10.1186/1471-2164-15-806) contains supplementary material, which is available to authorized users.

## Background

*Toxoplasma gondii* is an obligate intracellular parasite that can infect any nucleated cell of warm-blooded animals. The parasite has both sexual and asexual cycles where the sexual cycle takes place in the intestinal cells of the definitive feline host and the asexual cycle occurs in all warm-blooded animals [1]. The asexual stages of *T. gondii* consist of the rapidly replicating tachyzoite and the slow growing encysted bradyzoite. In the host, the tachyzoite is the prominent stage during initial acute infection [2]. Once the tachyzoite is subjected to stress from the host immune response, it differentiates to the bradyzoite form and eventually establishes a chronic infection [3]. The bradyzoite persists for the lifetime of the host as intracellular cysts present in striated muscle and the central nervous system [4].

*T. gondii* is one of the most prominent parasites in humans with prevalence rates between 10 and 80 percent worldwide, depending on the country [5]. Complications such as hydrocephaly, retinochoroiditis, mental retardation and even death can occur in developing fetuses [6, 7]. In immunocompetent humans, infection with *T. gondii* is generally asymptomatic presenting flu-like symptoms in approximately 10 percent of individuals [8]. Patients with compromised immune systems, such as those infected with HIV, are at great risk of developing severe symptoms such as Toxoplasmic Encephalitis (TE) and ocular infection that may result in blindness [9, 10]. Disease is largely associated with sporadic reactivation of the latent bradyzoite back to the rapidly replicating tachyzoite [11]. Currently there are no drugs that can combat the bradyzoite form of the parasite or effective vaccines to protect against infection. These issues highlight the critical need to understand the cellular triggers that control development between the tachyzoite and bradyzoite stages.

Previous work to characterize the transcriptome of tachyzoite and bradyzoites from *T. gondii* has primarily used microarray technology from samples prepared in tissue culture [12–14]. Transcriptomic studies have also used a combination of in vitro and in vivo samples with in vitro tachyzoites, in vivo bradyzoites, and oocysts collected from infected felines as well as tissue culture tachyzoites and bradyzoites, developing oocysts, and bradyzoites purified from mouse brains 21 days post-infection [12, 15] While these studies have provided valuable insight into this developmental process, the information that can be extracted is limited because tissue culture conditions for tachyzoite and bradyzoite development do not precisely model animal infections. Other in vivo microarray studies have compared peritoneal-derived tachyzoites from different strain types of *T. gondii* from wild type and interferon-γ (IFN-γ) deleted mice, and transcriptional changes in the brain of mice eight days after *T. gondii* infection [16–18]. These studies have highlighted important aspects of the developmental process, but the dynamic range of microarrays is restricted and sample preparations are unable to be simultaneously processed and analyzed for both host and pathogen. One way to overcome these limitations is RNA sequencing (RNA-seq), a breakthrough molecular tool that can provide the transcript profile (transcriptome) of total cellular RNA with a large dynamic range and improved sensitivity [19]. RNA-seq has detected novel *T. gondii* tachyzoite transcripts and alternative splicing between strains [20]. RNA-seq of tissue culture-derived bradyzoites has shown dysregulation of bradyzoite genes in the deletion mutant of a mucin domain containing cyst wall protein CST1 [21]. Using RNA-seq, transcriptome analysis has been performed to compare mouse brains that were uninfected or infected with *T. gondii* for 32 days [22]. These data highlight the sensitivity and depth of knowledge that can be obtained from RNA-seq studies; however, a time course of *T. gondii* infection and simultaneous analysis of the parasite transcriptome has not been performed.

To provide a more comprehensive analysis of *T. gondii* and the host during both acute and chronic infection, we collected RNA-seq data from three experimental groups of mice: uninfected, 10 and 28 days post-infection. Because *T. gondii* preferentially establishes cysts in the brains of mice and reactivation of cysts is the main cause of TE, we chose to analyze the brains of mice. A novel aspect of this dataset is that parasites were not purified from the brain tissue but instead, samples were rapidly processed so that RNA-seq reads represent the "interactome" between host and pathogen during the peak of acute and chronic infection. We report that many genes involved in *T. gondii* transcription, translation and metabolism are highly expressed during chronic infection. For the host, we find that more genes are increased in abundance during chronic versus (vs) acute infection, attesting to a continuously active host response even at 28 days post-infection.

## Results

### Sequencing and mapping the *T. gondii*/host interactome

**Figure 1:**
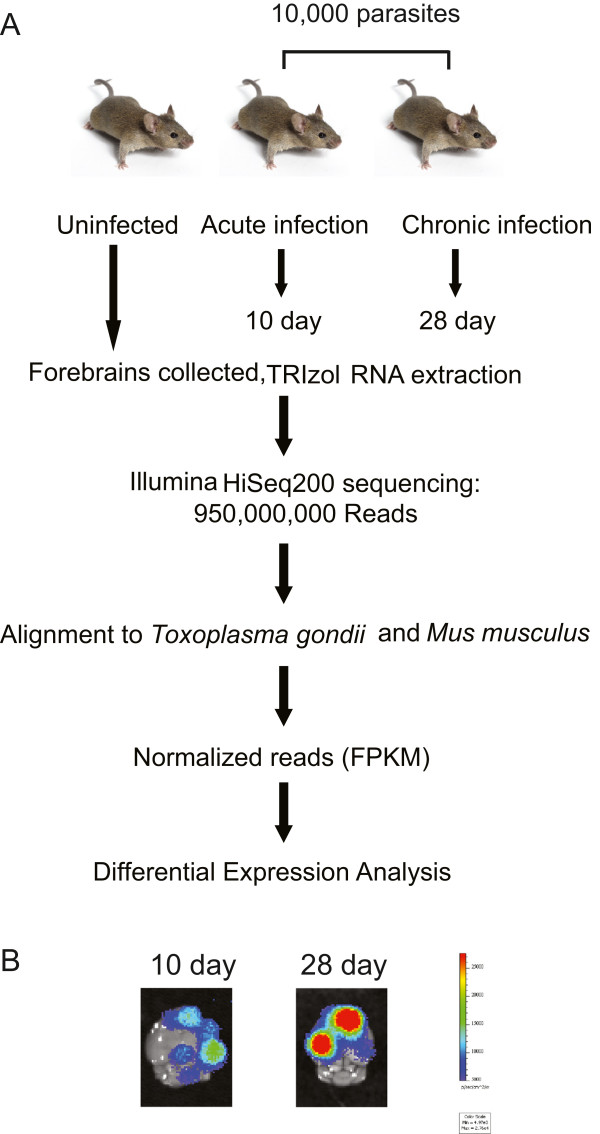
**Schematic of** ***T. gondii*** **/host dataset generation. (A)** Nine mice were divided into three experimental groups: uninfected, 10 days post-infection, and 28 days post-infection. Infected mice were given 10,000 type II ME49 parasites and sacrificed on the corresponding days. The forebrains were removed and homogenized in TRIzol, and RNA was extracted and purified. A cDNA library was generated from the RNA prior to IlluminaHiSeq2000 sequencing. Raw reads were aligned to either the *T. gondii* or *M. musculus* genomes, normalized and analyzed for differential gene expression. **(B)** To examine *T. gondii* in the brains of mice at the designated time points, mice were infected with 10,000 parasites of a bioluminescent *T. gondii*[62]. Shown are representative brains for 10 and 28 day post-infection mice, after the brains were soaked for 5 minutes in luciferin prior to imaging.

To study parasite*-*host gene expression dynamics we used RNA-seq on samples collected from forebrains of mice infected with type II strains of *T. gondii* (schematized in Figure 1). Type II strains of *T. gondii* have been detected in the mouse brain as early as 4 days post-infection and numbers continue to increase until 10 days post-infection [23, 24], the peak of acute infection [25]. Cyst structures are present in the brains of mice at 21 days post-infection [26], which is generally considered to be the beginning of chronic infection. By 28 days post-infection, cysts have stably formed in the brain while parasite numbers have decreased elsewhere in the body [23, 27]. When brains were sectioned and analyzed for parasite distribution, high numbers of *T. gondii* were observed in the frontal lobe at 32 days post-infection [22]. We examined mouse brains at 10 and 28 days post-infection using an In Vivo Imaging System (IVIS), which confirmed that parasites were primarily localized in the forebrains of mice (Figure 1). Therefore to maximize parasite transcripts as well as to compare the same host tissue during acute and chronic infection, we chose to collect mouse forebrain samples at 10 and 28 days post-infection as well as uninfected mice. Nine mouse forebrains were sequenced individually: three uninfected, three infected for ten days and three infected for 28 days. Nearly one billion 100 base-pair (bp) paired-end RNA sequences were generated. Between 81,000,000 and 114,000,000 reads were obtained from each forebrain sample (Table 1). Between 69-76% of the reads aligned to the *M. musculus* reference genome while approximately 0.1% aligned to the *T. gondii* TGME49 reference. Since *T. gondii* was not purified from the forebrains as a means to rapidly process the samples and preserve the interactome, uninfected mouse forebrain samples were mapped to the *T. gondii* TGME49 reference to determine the extent of false positive reads. A small number of uninfected mouse reads aligned to the *T. gondii* genomic reference file (Table 1). The reads aligned to *T. gondii* ribosomal associated RNA or small (~200-300 bp) hypothetical proteins, none of which were considered differentially expressed and were treated as background.

**Table 1:** **Mapped paired-end reads of individual mouse forebrain samples**

Days post-infection	Sample number	Total number of reads	mapped ***T. gondii***paired reads	mapped mouse paired reads
Uninfected	1	112075860	10752	71901643
Uninfected	2	112948998	7272	72770895
Uninfected	3	103209252	12175	69295619
10	1	102581171	51734	68040784
10	2	125546828	51788	81691030
10	3	81630704	21234	47283570
28	1	103765423	90414	65891584
28	2	113094439	61481	69756643
28	3	114177191	89520	71828085

The first column is the time-point during *T. gondii* infection for the mouse forebrain samples used for RNA extraction. As three replicates were used for each time point, the second column is the sample number. The third column is the total number of reads obtained from Illumina HiSeq sequencing. The fourth column is the total number of paired-end reads from each mouse forebrain sample that mapped to the *T. gondii* genomic reference file. The fifth column is the total number of paired-end reads from each experimental group that mapped to the *M. musculus* genomic reference file.

### Transcript abundance of *T. gondii*during acute and chronic infection in mice

Abundance estimates for each *T. gondii* gene were calculated and the three biological replicates for each time point were averaged before differential expression analysis was performed using Cuffdiff, a Cufflinks program. Cuffdiff calculates the fold change to determine which genes are differentially regulated between time points. Cuffdiff also calculates a p-value and q-value to determine if the fold change is significant. Each forebrain was treated as a biological replicate in Cuffdiff and therefore variation between replicates was considered when assigning a p-value. A p-value and q-value <0.05 were considered significant. It is important to note that parasites were not purified from mouse forebrains prior to RNA extraction. As a result, the concentration of *T. gondii* RNA could not be normalized between time points. To address the potential difference in parasite numbers between experimental time points quantitative PCR was performed using genomic DNA extracted from the forebrains at the time of RNA extraction. Using a standard curve generated from serial dilutions of genomic DNA extracted from a known number of parasites, we found approximately 500 parasites per 350 ng of genomic DNA in 10 day post-infection samples and approximately 1500 parasites per 350 ng of genomic DNA in the forebrains of mice infected for 28 days. These numbers are in agreement with the average number of reads generated from each time point with approximately twice as many reads aligning to the *T. gondii* genome in the 28 day samples than the 10 day samples (Table 1). To further examine differences in parasite numbers between acute and chronic infection samples, the FPKM fold change of housekeeping genes α-tubulin, actin, glyceraldehyde 3-phosphate dehydrogenase 1 and 2 (GAPDH 1 and 2), and hexokinase were examined between acute and chronic time points (Table 2). The fold change of these housekeeping genes between chronic and acute time points were 0.6-1.8, suggesting that global parasite transcript levels in our acute and chronic infection samples do not dramatically change.

**Table 2:** **Fold change between chronic and acute infection for previously characterized** ***T. gondii*** **genes**

Gene ID	Description	Fold change: chronic/acute
TGME49_316400	α tubulin	0.6
TGME49_209030	actin	1.2
TGME49_289690	GAPDH 1	1.8
TGME49_269190	GAPDH 2	1
TGME49_265450	hexokinase	0.7
TGME49_291890	MIC1	0.024
TGME49_233460	SAG1	0.0055
TGME49_259020	BAG1	48
TGME49_268860	ENO1	38

First column is the gene number from ToxoDB.org. The middle column is gene description where α-tubulin, actin, hexokinase, GAPDH-1 and 2 are housekeeping genes, tachyzoite specific genes are SAG1 and MIC1, and bradyzoite specific genes are BAG1 and ENO1. The third column is the average FPKM value for chronic genes divided by the average FPKM value for acute genes.

When comparing the fold change of previously characterized tachyzoite-specific genes, surface antigen 1 (SAG1) and microneme protein (MIC) 1, and bradyzoite specific genes bradyzoite antigen 1 (BAG1) and enolase 1 (ENO1), we saw large differential expression between acute and chronic infected mice (Table 2). These results along with qPCR performed on genomic DNA and quantitation of the reads that map to the *T. gondii* genome (Table 1) suggest that the significant differential expression of genes between time points is not due to an overabundance of transcripts in chronically infected mice compared to acutely infected mice. This data is supported in previous work that determined there is minimal increase in parasite numbers in the brain between 10 and 20 days [28]. To account for minimal variability of parasite numbers at 28 days post-infection differentially expressed *T. gondii* genes with a fold-change of >5 were considered for further analysis. The low abundance of SAG-1 and high abundance of BAG-1 between 28 day post-infection and 10 day post infection time points also suggests that tachyzoites are the primary stage present at 10 days post-infection, while the majority of parasites at 28 days post-infection are in the bradyzoite stage.

**Figure 2:**
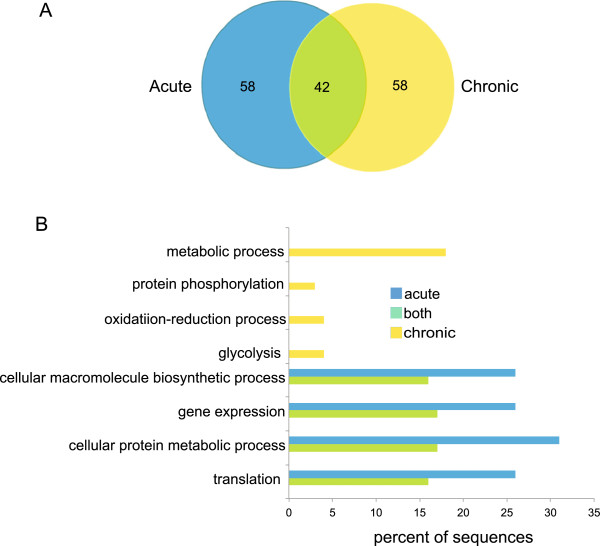
**Top 100** ***T. gondii*** **genes from acute and chronic infection. (A)** Venn diagram compares the 100 *T. gondii* genes with the greatest FPKM value from acute and chronic time points. Of the top 100 genes for each stage, 42 genes were similarly abundant (green), and 58 were only abundant in acute (blue) or chronic (yellow) infection. **(B)** GO terms were assigned to the top 100 *T. gondii* genes from acute and chronic infection. Genes were grouped based on whether they are similar or different between time points prior to GO term analysis.

To explore the similarities in expression of *T. gondii* genes during acute and chronic infection, the 100 most highly expressed genes from acute and chronic time points were compared. Forty-two of the most highly expressed *T. gondii* genes during the acute stage were also among the 100 most highly expressed transcripts during the chronic stage (Figure 2A). To help interpret the biological functions of these genes, statistically over-represented GO terms were compiled (Figure 2B) using the Blast2GO program, a GO term analysis program for non-model organisms [29]. The GO term categories for the genes with the greatest abundance in both acute and chronic samples were transcription, translation, macromolecule biosynthesis and cellular metabolism.

### Differential expression of *T. gondii*between acute and chronic infection in mice

To further investigate the transcriptome of *T. gondii* during acute and chronic infection, differential expression analysis was performed. Fold change and significance values were calculated for each of the ~8900 annotated *T. gondii* genes (Additional file 1). From this analysis, we found 547 significantly differentially expressed genes (DEGs, with p-value and q-value <0.05) between acute and chronic infection. Of these, the DEGs with a fold change of >5 are presented in Tables 3 and 4.

**Table 3:** ***T. gondii*** **DEGs that were more abundant >5-fold in acute vs chronic infection**

Gene ID	Description	Fold change: acute/chronic
TGME49_233480	SAG-related sequence SRS29C (SRS29C)	305
TGME49_233460	SAG-related sequence SRS29B (SAG1)	181
TGME49_291890	microneme protein MIC1 (MIC1)	41
TGME49_241240	hypothetical protein	40
TGME49_224460	aminopeptidase	26
TGME49_262050	rhoptry kinase family protein ROP39	23
TGME49_297880	dense granule protein DG32	19
TGME49_271050	SAG 34A/SAG/2	18
TGME49_294200	glucose-6-phosphate 1-dehydrogenase	13
TGME49_230160	hypothetical protein	12
TGME49_261740	hypothetical protein	12
TGME49_291960	rhoptry kinase family protein ROP40	10
TGME49_268850	enolase 2	9
TGME49_277490	hypothetical protein	9
TGME49_215960	hypothetical protein	9
TGME49_222170	dense-granule antigen DG32	8
TGME49_293430	hypothetical protein	8
TGME49_262730	rhoptry protein ROP16 (ROP16)	8
TGME49_314400	pyruvate dehydrogenase E1 component	8
TGME49_315320	SAG-related sequence SRS52A (SRS52A)	7
TGME49_200360	hypothetical protein	7
TGME49_285870	SAG-related sequence SRS20A (SRS20A)	7
TGME49_269950	hypothetical protein	7
TGME49_247280	hypothetical protein	7
TGME49_294800	elongation factor 1-alpha (EF-1-ALPHA)	7
TGME49_213050	hypothetical protein	6
TGME49_249180	bifunctional dihydrofolate reductase-thymidylate synthase	6
TGME49_226710	hypothetical protein	6
TGME49_237880	hypothetical protein	6
TGME49_250115	hypothetical protein	6
TGME49_254720	dense granule protein GRA8 (GRA8)	6
TGME49_253930	GCC2 and GCC3 domain-containing protein	6
TGME49_299780	hypothetical protein	6
TGME49_275860	hypothetical protein	6
TGME49_310780	dense granule protein GRA4 (GRA4)	6
TGME49_277080	microneme protein MIC5 (MIC5)	6
TGME49_226960	phosphofructokinase PFKII (PFKII)	6
TGME49_243730	rhoptry protein ROP9 (ROP9)	6
TGME49_207400	hypothetical protein	6
TGME49_259240	ribosomal protein RPS21 (RPS21)	6
TGME49_205340	ribosomal protein RPS12 (RPS12)	6
TGME49_209150	non-proton pumping type-II NADH dehydrogenase I (NDH2-I)	6
TGME49_253690	hypothetical protein	5
TGME49_229670	ribosomal protein RPS23 (RPS23)	5
TGME49_244690	hypothetical protein	5
TGME49_212290	ribosomal protein RPS19 (RPS19)	5
TGME49_288360	tryptophanyl-tRNA synthetase (TrpRS2)	5
TGME49_251810	translation initiation factor eIF-5A	5
TGME49_200350	subtilisin SUB3 (SUB3)	18*
TGME49_250955	KRUF family protein	21*
TGME49_279350	hypothetical protein	23*
TGME49_313250	hypothetical protein	26*
TGME49_307760	Tubulin-tyrosine ligase family protein	27*
TGME49_315740	SAG-related sequence SRS54	27*
TGME49_243700	hypothetical protein	29*
TGME49_293210	hypothetical protein	30*
TGME49_276110	cytochrome b5 family heme/steroid binding	34*
TGME49_218740	membrane protein	65*
TGME49_294805	hypothetical protein	77*
TGME49_294990	hypothetical protein	106*
TGME49_216770	hypothetical protein	125*
TGME49_230180	hypothetical protein	129*
TGME49_305050	calmodulin	295*

First column is the gene number from ToxoDB.org. The second column is the gene description. The far right column is the average FPKM value from acute infection divided by the average FPKM value from chronic infection, called the fold change. * in the fold change column indicates the average FPKM value during chronic infection was 0, and could not be divided by the average FPKM value in acute samples. A p-value and q-value of <0.05 was considered to be statistically significant and only genes that met these standards were included on this table.

**Table 4:** ***T. gondii*** **DEGs that were more abundant >5 fold in chronic vs acute infection**

Gene ID	Description	Fold change: chronic/acute
TGME49_224630	zinc finger (CCCH type) protein	86
TGME49_259020	bradyzoite antigen BAG1 (BAG1)	48
TGME49_202020	DnAK-TPR	44
TGME49_278080	Toxoplasma gondii family A protein (SUSA-1)	32
TGME49_291040	lactate dehydrogenase LDH2 (LDH2)	31
TGME49_200250	microneme protein MIC17A (MIC17A)	29
TGME49_260190	microneme protein MIC13 (MIC13)	29
TGME49_267680	microneme protein MIC12 (MIC12)	28
TGME49_262970	hypothetical protein	26
TGME49_245530	hypothetical protein	26
TGME49_204420	oocyst wall protein OWP1 (OWP1)	23
TGME49_318880	hypothetical protein	18
TGME49_289370	hypothetical protein	18
TGME49_207210	hypothetical protein	18
TGME49_209985	cAMP-dependent protein kinase	18
TGME49_309930	melibiase subfamily protein	17
TGME49_320260	hypothetical protein	15
TGME49_293780	hypothetical protein	14
TGME49_216140	tetratricopeptide repeat-containing protein	14
TGME49_280570	SAG-related sequence SRS35A (SRS35A)	13
TGME49_320190	SAG-related sequence SRS16B (SRS16B)	11
TGME49_250940	hypothetical protein	11
TGME49_306620	AP2 domain transcription factor AP2IX-9 (AP2IX9)	11
TGME49_209755	hypothetical protein	11
TGME49_207160	SAG-related sequence SRS49D (SRS49D)	10
TGME49_202030	hypothetical protein	10
TGME49_312600	heat shock protein HSP21 (HSP21)	9
TGME49_290000	hypothetical protein	9
TGME49_256760	pyruvate kinase PyK1 (PYKI)	8
TGME49_225290	GDA1/CD39 (nucleoside phosphatase)	7
TGME49_269670	hypothetical protein	7
TGME49_253330	Rhoptry kinase family protein	7
TGME49_225540	hypothetical protein	7
TGME49_282130	hypothetical protein	6
TGME49_207710	phosphatidylinositol synthase	6
TGME49_205680	hypothetical protein	6
TGME49_276200	hypothetical protein	6
TGME49_283780	glucose-6-phosphate isomerase GPI (GPI)	6
TGME49_285980	glucosephosphate-mutase GPM1 (GPM1)	6
TGME49_264420	lipoprotein	6
TGME49_226420	peptidase family M3 protein	6
TGME49_290980	glycine C-acetyltransferase	6
TGME49_275320	penicillin amidase	5
TGME49_201840	aspartyl protease ASP1 (ASP1)	5
TGME49_246080	NAD dependent epimerase/dehydratase	5
TGME49_222370	SAG-related sequence SRS13 (SRS13)	5
TGME49_315760	AP2 domain transcription factor AP2XI-4 (AP2XI4)	5
TGME49_294400	hypothetical protein	5
TGME49_215910	hypothetical protein	5
TGME49_224950	calcium-dependent protein kinase CDPK5 (CDPK5)	5
TGME49_256060	nucleosome assembly protein (nap) protein	5
TGME49_205750	histone deacetylase complex subunit Sin3 (SIN3)	5
TGME49_253320	hypothetical protein	15*
TGME49_254330	lipase	16*
TGME49_309790	hypothetical protein	16*
TGME49_269300	lipase	17*
TGME49_223258	hypothetical protein	17*
TGME49_207875	GCC2 and GCC3 domain-containing protein	17*
TGME49_269020	hypothetical protein	18*
TGME49_261200	TBC domain-containing protein	18*
TGME49_254150	hypothetical protein	18*
TGME49_306500	hypothetical protein	18*
TGME49_245440	hypothetical protein	19*
TGME49_207980	PIG-P protein	19*
TGME49_220150	50S ribosomal protein L16	19*
TGME49_320720	hypothetical protein	19*
TGME49_268765	hypothetical protein	19*
TGME49_260530	Sel1 repeat-containing protein	22*
TGME49_269320	hypothetical protein	23*
TGME49_215300	hypothetical protein	24*
TGME49_308096	rhoptry kinase family protein	25*
TGME49_240470	hypothetical protein	25*
TGME49_460810	ribosomal RNA	25*
TGME49_310045	hypothetical protein	26*
TGME49_224180	hypothetical protein	26*
TGME49_205210	hypothetical protein	26*
TGME49_204040	hypothetical protein	27*
TGME49_297850	Branched-chain-amino-acid aminotransferase	27*
TGME49_215130	adaptor-related protein complex 3	27*
TGME49_207600	tubulin/FtsZ family, GTPase	27*
TGME49_200230	microneme protein MIC17C (MIC17C)	28*
TGME49_231125	hypothetical protein	30*
TGME49_219610	hypothetical protein	31*
TGME49_240480	cpw-wpc domain-containing protein	31*
TGME49_315520	calcium binding egf domain-containing protein	32*
TGME49_232430	hypothetical protein	33*
TGME49_244412	hypothetical protein	33*
TGME49_223855	RNA recognition motif-containing protein	35*
TGME49_260325	hypothetical protein	37*
TGME49_234625	EGF family domain-containing protein	37*
TGME49_255460	hypothetical protein	40*
TGME49_321710	IgA-specific serine endopeptidase	43*
TGME49_319090	proteasome maturation factor ump1 protein	50*
TGME49_209090	hypothetical protein	52*
TGME49_210682	hypothetical protein	53*
TGME49_213480	hypothetical protein	53*
TGME49_210095	hypothetical protein	57*
TGME49_313890	hypothetical protein	58*
TGME49_271450	hypothetical protein	59*
TGME49_321700	hypothetical protein	80*
TGME49_219742	hypothetical protein	87*
TGME49_266600	Kazal-type serine protease inhibitor	89*
TGME49_257970	hypothetical protein	89*
TGME49_258370	rhoptry kinase family protein ROP28 (ROP28)	103*
TGME49_295662	hypothetical protein	110*
TGME49_278882	GDA1/CD39 (nucleoside phosphatase)	113*
TGME49_264150	hypothetical protein	141*

First column is the gene number from ToxoDB.org. The far right column is the fold change calculated from the average FPKM value from chronic infection divided by the average FPKM value from acute infection. * in the fold change column indicates the average FPKM value during acute infection was 0, and could not be divided by the average FPKM value in chronic infection. A p-value and q-value of <0.05 was considered to be statistically significant and only genes that met these standards were included on this table.

Sixty-three *T. gondii* genes were >5 fold more abundant in acute compared to chronic infection (Table 3). SAG-related sequence (SRS) are a family of GPI-anchored surface antigens related to the first characterized *T. gondii* surface antigen, SAG1 [30]. SRS2/SRS29C was the most differently expressed gene in acute compared to chronic infection, 305-fold. Five additional SRS genes were >5 fold more abundant: SRS20A, SAG1/SRS29B, SAG2/SRS34A, SRS54 and SRS52A. Four genes for rhoptry proteins (ROP) were >5 fold more abundant during acute infection: ROP9, ROP16, ROP39, and ROP40. ROP16 is involved in decreased synthesis of cytokines in mouse bone marrow-derived macrophages [31]. ROP9 is a tachyzoite-specific protein with no known function [32]. ROP39 and ROP40 have homology to ROP2, but the functions have yet to be elucidated. Because of the extensive study of tachyzoites, most of the DEGs highly abundant in acute compared to chronic infection (Table 3) have been previously identified as tachyzoite-specific markers. Twenty-six of the 63 acute infection DEGs encode hypothetical proteins, with no homology to any annotated protein in the BLAST database. Characterizing these hypothetical proteins could be vital to understanding parasites during acute infection.

Fifty-one of the 107 *T. gondii* DEGs associated with chronic infection with a >5 fold change are annotated as hypothetical (Table 4). Among the chronic infection DEGs, four microneme proteins were identified MIC12, MIC13, MIC17A and MIC17C. MIC12 and MIC13 were previously shown to be bradyzoite specific [12], but our data revealed MIC17A and MIC17C abundant specifically during chronic infection. Another interesting group of chronic infection DEGs are those involved in glycolysis: glucose-6-phosphate isomerase, pyruvate kinase, lactate dehydrogenase 2, and glucosephosphate mutase. Previous data showed that tachyzoites and bradyzoites use the glycolytic pathway differently with lactate dehydrogenase 2 and pyruvate kinase being up-regulated during the bradyzoite stage [33, 34]. Our data shows that in addition to these two previously described bradyzoite-specific glycolytic enzymes, glucose-6-phosphate isomerase and glucosephosphate mutase are also more abundant in chronic vs acute infection. These results strengthen the idea that bradyzoites do not have an active TCA cycle because the transcripts of the key enzymes in the TCA cycle were less abundant in chronic infection compared to acute. Four SRS genes were also identified as more abundant in chronic compared to acute infection. SRS35A, also known as SAG4 and P18, is a long-known bradyzoite-specific marker [35]. SAG2C/SRS49D and SRS9/SRS16B are previously identified bradyzoite-specific genes that are important for persistence of infection [36–39]. SRS13 was identified as up-regulated in a microarray analysis comparing tissue culture tachyzoites to mouse-derived bradyzoites, but the function has yet to be determined [12]. Several novel chronic infection DEGs were found including DnAK-TPR, heat-shock protein 21, calcium dependent protein kinase CDPK5 (Table 4). The high number of novel and hypothetical DEGs highlights the fact that much is still unknown about *T. gondii* during animal infection. These hypothetical proteins have no known homology to proteins in the host and therefore could be essential and specific to parasite function during infection. Identification of novel DEGs that could play critical roles in *T. gondii* infection is the first step in elucidating potential targets for both vaccine and drug development.

To highlight the accuracy and sensitivity of RNA-seq, q-PCR was performed on a family of CCCH zinc fingers in *T. gondii* that are more abundant in our data set during chronic infection. TGME49_224630, TGME49_262970, and TGME49_311100 contain CCCH zinc finger domains and had a fold change of 86, 26, and 4.7, respectively (Table 4 excluding TGME49_311100 which did not meet the >5 fold cut-off). These genes were chosen based on the possible similarity in function and the range of transcript differential expression between chronic and acute infection. The increase in abundance of these transcripts between 10 day and 28 day post-infection was observed using qPCR when normalized to the house keeping gene tub1a. The fold change between chronic and acute infection for TGME49_224630, TGME49_262970, and TGME49_311100 were 53, 79, and 6.1 respectively. Not only does this data demonstrate the range and accuracy of RNA-seq, but also confirms the validity of the differential expression analysis.

### Differential expression of host genes during *T. gondii*infection

**Figure 3:**
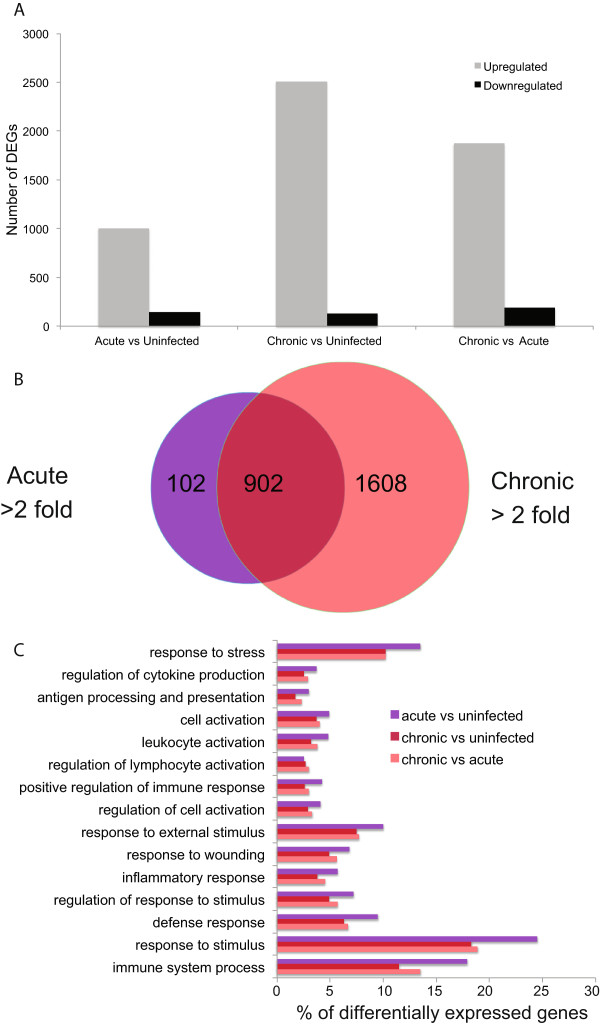
**More host genes have increased abundance during chronic infection. (A)** DEGs in the mouse with a fold change >2 were grouped based on increased abundance (grey) and decreased abundance (black) between acute vs uninfected, chronic vs uninfected, and chronic vs acute time points. **(B)** A Venn diagram was created to compare DEGs with increased abundance in acute vs uninfected (purple) and chronic vs uninfected time points (red). Of the 1004 more abundant DEGs in acute vs uninfected, 902 were also more abundant in the chronic vs uninfected group (magenta). **(C)** To explore the function of DEGs analyzed in the Venn diagram, a GO term enrichment analysis was performed.

To understand the transcriptional changes of the host during stages of *T. gondii* infection, differential expression analysis was conducted. Differential expression was determined between acute vs uninfected, chronic vs uninfected, and acute vs chronic time points. Genes were considered differentially expressed if the p-value and q-value was <0.05 and the fold change between time points was >2-fold. The host underwent extensive transcriptional changes during *T. gondii* infection (Figure 3A). When comparing acute *T. gondii* infected mice with uninfected, 1004 mouse genes were more abundant during acute infection and 143 were less abundant (Figure 3A). Over twice as many mouse genes, 2510, were more abundant in mice with a chronic *T. gondii* infection compared to uninfected while only 132 genes were less abundant. Finally, 1872 mouse genes were more abundant and 190 were less abundant in chronically vs. acutely infected mice. This increase of differentially regulated host genes during chronic infection is illustrated by Venn diagram analysis of DEGs >2-fold (Figure 3B). To understand the similarities and differences between DEGs more abundant during acute vs uninfected and chronic vs uninfected time points, we identified genes that had increased abundance at both time points as well as those found only in acute or chronic (Additional files 2, 3 and 4). Out of the 1004 DEGs more abundant in acute vs uninfected, 902 were also abundant in the chronic vs uninfected group. More mouse genes, 1608, were increased in abundance in the chronic vs uninfected time point that were not considered differentially expressed in acute. This data suggests genes activated during the peak of acute infection are maintained during chronic infection and that an entirely new subset of transcripts are expressed during chronic infection*.* The increase in differentially expressed genes during chronic infection could also be due to recruitment of cells to the site of infection.

### GO term enrichment analysis of DEGs in mice during *T. gondii*infection

To understand the functions of DEGs in mice during acute and chronic *T. gondii* infection and to characterize the overlap between DEGs at these time points (Figure 3B) we performed GO term enrichment analyses. Among the host genes more abundant during both acute and chronic infection stages, overrepresented GO terms were related to stress and immune responses (Figure 3C). This suggests infection with *T. gondii* stimulates the immune response and/or immune cell recruitment into the brain even when the majority of parasites are in the encysted stage, as seen by the increased abundance of BAG1 and decreased abundance of SAG1 (Table 2). It also suggests a specific subset of host genes are responsible for immune stimulation during chronic infection that are distinct from acute, although many acute infection associated genes are still activated.

**Figure 4:**
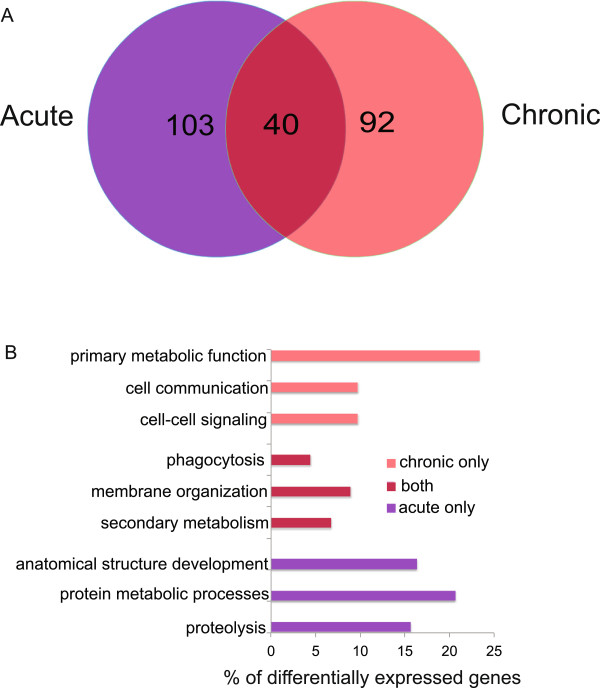
**Analysis of host genes with decreased abundance during** ***T. gondii*** **infection. (A)** A Venn diagram was created to compare DEGs >2-fold decreased abundance in acute vs uninfected (purple) and chronic vs uninfected (red) mice. Forty host genes were less abundant during both acute and chronic infection (magenta). **(B)** To explore the function of DEGs analyzed in the Venn diagram, a GO term enrichment analysis was performed. Functionally enriched GO terms display little similarity between time points.

Only a small number of host DEGs were less abundant during both acute and chronic infection (Figure 4A). Go term analysis of DEGs with decreased abundance showed little functional overlap between the different stages of infection*.* The few commonalities in GO categories included secondary metabolism, membrane organization and phagocytosis (Figure 4B). The proposed functions of the DEGs that were less abundant specifically during acute infection were different from those less abundant during chronic infection. GO terms for genes with decreased abundance specifically during acute infection were proteolysis, protein metabolic process, and anatomical structure, while GO terms specific to chronic infection were cell-cell communication and primary metabolic function. The enrichment of metabolism-associated processes among less abundant DEGs suggests a link between *T. gondii* infection and host metabolism, possibly as a means of restricting parasite growth.

### Analysis of the mouse genes with increased abundance during *T. gondii*infection

**Figure 5:**
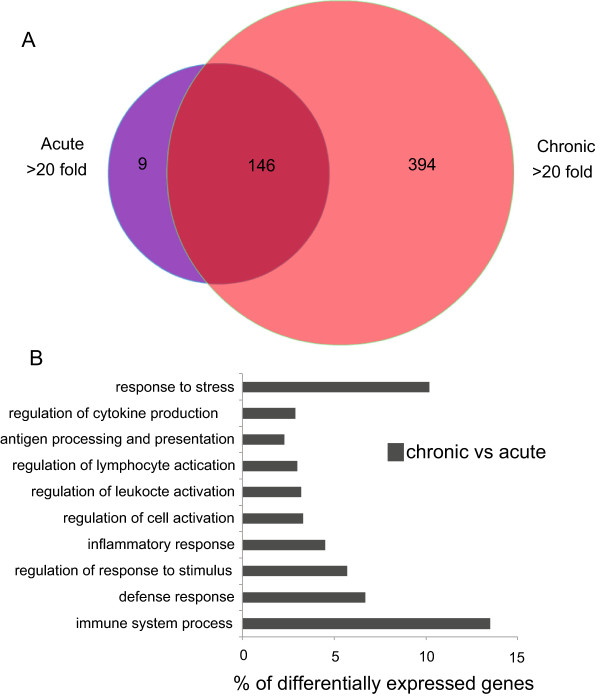
**Few highly expressed DEGs are specific to acute infection. (A)** To better characterize abundant DEGs, a Venn diagram of the more abundant DEGs in the mouse with a fold change >20 between acute vs uninfected (purple) and chronic vs uninfected (red) time points. Of these more abundant DEGs, 146 were shared between acute and chronic infection (magenta). Only 9 DEGs had a fold change >20 in acute vs uninfected that were also not highly abundant in chronic vs uninfected time points. **(B)** To analyze the function of the host more abundant DEGs in chronic vs acute time points, GO term enrichment analysis was performed. Many genes differentially expressed in chronic infection are associated with immune regulation and stress response.

Given that more host transcripts had >2-fold increased abundance during chronic infection (Figure 3B), further examination of the most abundant mouse genes during acute and chronic time points was performed to enhance the understanding of the host response. DEGs with a FPKM fold change >20 were compared between acute vs uninfected and chronic vs uninfected mice (Figure 5A). 155 genes met this cut-off in the acute vs uninfected group while 540 genes had 20-fold or higher FPKM values in chronic vs uninfected time points. Of these more abundant DEGs, 146 were shared between acute vs uninfected and chronic vs uninfected groups. Only 9 more abundant DEGs had a fold change >20 in acute vs uninfected that were not highly abundant in chronic vs uninfected time points. Several of these acute-infection specific host genes belong to the family of guanylate-binding proteins, which are GTPases that are induced by interferon-γ (Table 5). Conversely, 394 DEGs had a fold change of >20 in chronic vs uninfected that did not meet this cut-off in acute vs uninfected. The majority of genes increased in abundance during acute infection are maintained into chronic infection. These results show that few host genes are specifically increased during acute *T. gondii* infection. It also suggests a unique set of host genes are differentially expressed during chronic infection.

**Table 5:** **Mouse DEGs more abundant in acute, but not chronic infection**

Gene ID	Gene name	Description	Fold change (Acute/uninfected)	Fold change (Chronic/uninfected)
ENSMUSG00000088071	Gm22818	predicted gene, 22818	276	1
ENSMUSG00000002831	Gbp10	guanylate-binding protein 10	183	No transcripts in Chronic
ENSMUSG00000002833	Gbp6	guanylate binding protein 6	130	No transcripts in Chronic
ENSMUSG00000024334	Gbp4*	guanylate binding protein 4	61	No transcripts in Chronic
ENSMUSG00000075010	Gbp8*	guanylate-binding protein 9	61	No transcripts in Chronic
ENSMUSG00000085377	Gbp9*	guanylate-binding protein 8	61	No transcripts in Chronic
ENSMUSG00000098049	BC042782^#^	cDNA sequence BC042782	52	1.8
ENSMUSG00000079362	n-R5s189^#^	nuclear encoded rRNA 5S 189	52	1.8
ENSMUSG00000005800	Plin4	perilipin 4	27	1

The first column is the ensemble gene ID and the second is the gene name. The third column is the official gene symbol from ensemble.org, and the fourth column is the fold change as average acute FPKM value was divided by uninfected. The fifth column is the fold change as average chronic FPKM value was divided by uninfected. Only genes with a p-value and q-value <0.05 were considered differentially expressed. * and ^#^ mark genes located in the same contiguous loci and thus reads mapping to this area were combined.

### Elevation of immune response genes during chronic infection

To assess the function of the mouse genes increased in abundance specifically during chronic infection, GO term enrichment was performed on genes with a fold change >20 in chronic vs acute infection. GO terms for genes highly increased in abundance during chronic infection were related to stress and immune responses (Figure 5B). Table 6 shows the top 50 DEGs more abundant during chronic infection. Many of these genes are immunoglobulin heavy chain variable regions, which share sequence similarity and can be difficult to differentiate between. The variable regions of immunoglobulins are responsible for specificity of antibodies to antigens, suggesting a different subset of antibodies being produced in response to *T. gondii* antigens that may not be abundant during acute infection. Another highly differentially expressed set of genes are H2-EB2 and H2-M2, both of which are involved in antigen presentation. H2-EB2 is an MHC class I membrane-associated protein while H2-M2 is an MHC class II membrane-associated protein. These antigen-presenting proteins are key players in the continued stimulation of the immune system at later time points. This increased in abundance of immune genes during chronic infection indicates a unique set of DEGs may be involved in the chronic infection immune response and/or a novel population of immune cells are recruited into the brain after acute infection*.*

**Table 6:** **The top 50 mouse DEGs more abundant in chronic vs. acute infection**

Gene ID	Description	Gene symbol	Fold change (chronic vs acute)
ENSMUSG00000076652	immunoglobulin heavy variable 7-3	Ighv7-3	672*
ENSMUSG00000076653	immunoglobulin heavy variable 7-2	Ighv7-2	672*
ENSMUSG00000095571	immunoglobulin heavy variable 5-17	Ighv5-17	672*
ENSMUSG00000076564	immunoglobulin kappa chain variable 12-46	Igkv12-46	656*
ENSMUSG00000096422	immunoglobulin kappa variable 12-44	Igkv12-44	656*
ENSMUSG00000076604	immunoglobulin kappa joining 1	Igkj1	353*
ENSMUSG00000076605	immunoglobulin kappa joining 2	Igkj2	353*
ENSMUSG00000076607	immunoglobulin kappa joining 4	Igkj4	353*
ENSMUSG00000076608	immunoglobulin kappa joining 5	Igkj5	353*
ENSMUSG00000076612	immunoglobulin heavy constant gamma 2C	Ighg2c	23*
ENSMUSG00000076617	immunoglobulin heavy constant	Ighm	23*
ENSMUSG00000076618	immunoglobulin heavy joining 4	Ighj4	23*
ENSMUSG00000076619	immunoglobulin heavy joining 3	Ighj3	23*
ENSMUSG00000076621	immunoglobulin heavy joining 1	Ighj1	23*
ENSMUSG00000094028	immunoglobulin heavy diversity 4-1	Ighd4-1	23*
ENSMUSG00000095079	immunoglobulin heavy constant alpha	Igha	23*
ENSMUSG00000095007	immunoglobulin kappa chain variable 12-41	Igkv12-41	356
ENSMUSG00000076609	immunoglobulin kappa chain, constant region	Igkc	353
ENSMUSG00000016283	histocompatibility 2, M region locus 2	H2-M2	111
ENSMUSG00000002992	apolipoprotein C-II	Apoc2	70
ENSMUSG00000074336	apolipoprotein C-IV	Apoc4	70
ENSMUSG00000019987	arginase, liver	Arg1	69
ENSMUSG00000071716	apolipoprotein L 7e	Apol7e	60
ENSMUSG00000058216	cDNA sequence BC021614	BC021614	45
ENSMUSG00000042262	chemokine (C-C motif) receptor 8	Ccr8	44
ENSMUSG00000014453	B lymphoid kinase	Blk	38
ENSMUSG00000050063	kallikrein related-peptidase 6	Klk6	38
ENSMUSG00000082976	predicted gene 15056; similar to beta-defensin 52	Gm15056	38
ENSMUSG00000053977	CD8 antigen, alpha chain	Cd8a	37
ENSMUSG00000030577	CD22 antigen; hypothetical protein LOC100047973	Cd22	36
ENSMUSG00000020017	histidine ammonia lyase	Hal	35
ENSMUSG00000027863	CD2 antigen	Cd2	35
ENSMUSG00000067149	immunoglobulin joining chain	Igj	35
ENSMUSG00000030724	CD19 antigen	Cd19	34
ENSMUSG00000067341	histocompatibility 2, class II antigen E beta2	H2-Eb2	34
ENSMUSG00000068129	cystatin F (leukocystatin)	Cst7	33
ENSMUSG00000079293	C-type lectin domain family 7, member a	Clec7a	32
ENSMUSG00000096594	immunoglobulin kappa variable 8-19	Igkv8-19	32
ENSMUSG00000002033	CD3 antigen, gamma polypeptide	Cd3g	31
ENSMUSG00000050232	chemokine (C-X-C motif) receptor 3	Cxcr3	28
ENSMUSG00000053044	CD8 antigen, beta chain 1	Cd8b1	28
ENSMUSG00000054672	RIKEN cDNA 5830411 N06 gene	5830411N06Rik	28
ENSMUSG00000094738	predicted gene, 26177	Gm26177	28
ENSMUSG00000005947	integrin alpha E, epithelial-associated	Itgae	27
ENSMUSG00000022657	CD96 antigen	Cd96	27
ENSMUSG00000024669	CD5 antigen	Cd5	27
ENSMUSG00000024910	cathepsin W	Ctsw	27
ENSMUSG00000031933	folate receptor 4 (delta)	Folr4	27
ENSMUSG00000026070	interleukin 18 receptor 1	Il18r1	26
ENSMUSG00000035042	chemokine (C-C motif) ligand 5	Ccl5	25

The first column is the ensemble gene ID and the second is the gene name. The third column is the official gene symbol from ensemble.org, and the fourth column is the fold change as average chronic FPKM value was divided by acute. Only genes with a p-value and q-value <0.05 were considered differentially expressed. * in the fold change column indicates the average FPKM value during acute infection was 0, and could not be divided by the average FPKM value in chronic infection.

## Discussion

Infection with *T. gondii* is often asymptomatic in immune competent individuals, but presents serious health risks if acquired congenitally or if a person becomes immune-compromised. Rising evidence is showing complications, such as psychiatric disorders and increased rates of suicide, occur in people with healthy immune systems who have a chronic *T. gondii* infection [40, 41]. Currently, drug treatment is only effective against the acute stage of infection and there are no therapeutics interventions available to target the encysted form during chronic infection. There are also no vaccines against *T. gondii* approved for use in humans. This lack of therapeutic intervention highlights the need to better understand the biological differences between the acute rapidly replicating form of the parasite and the chronic associated cyst stage.

Our data provides a list of candidate genes that could be targeted for novel therapeutics or gene deletion to create a non-persistent vaccine strain (Tables 3 and 4). Many of these genes highly differentially regulated are hypothetical proteins with no known orthologs in other organisms. Hypothetical *T. gondii* genes not found in the mammalian host could be excellent pathogen specific drug targets. The fact that multiple *T. gondii* microneme proteins are more abundant in chronic infection stages raises the question of whether parasites during chronic infection are actively invading cells. If parasites are invading at this time, this could explain the continued stimulation of the host response so late in infection. Although microarray analysis of human fibroblasts showed that tissue culture derived bradyzoites stimulated a weaker immune response than tachyzoites after two days [42]. An additional *T. gondii* gene that is more abundant in chronic infection is cAMP-dependent protein kinase. cAMP-dependent protein kinase is crucial for growth of tachyzoites and is proposed to be critical in tachyzoite to bradyzoite stage conversion [43, 44], but the mechanism is unknown. Another potentially interesting *T. gondii* gene that is more abundant in chronic infection is calcium dependent protein kinase 5, CDPK5. The *T. gondii* paralog, TgCDPK1, was shown to be necessary for tachyzoite motility, invasion and egress [45]. Studying the role of CDPK5 in bradyzoite development could be pivotal in understanding the biology of this stage.

Our study also provides crucial insight into host response to the parasite during both acute and chronic infection. It shows that, at least in the beginning stages of chronic infection, the host immune system is still actively combating infection. Chronic infection of *T. gondii* is typically thought of as a period in which the parasite transitions to the encysted, less immune stimulatory form resulting in a dampening of the immune response. Another study assessing the mouse transcriptome showed many immune associated genes are still expressed at 32 days post *T. gondii* infection [22]. To assess the overarching function of genes increased in abundance between acute and chronic infection Kyoto Encyclopedia of Genes and Genomes (KEGG) pathway analysis was performed (Additional file 5). The KEGG database is a bioinformatics tool that assembles large-scale molecular datasets, such as gene lists, into biological pathway maps (example Additional file 6). Analysis of our dataset suggests active NK cells are recruited to the brain during chronic infection by the increased abundance of perforin, granzymes A and B, and IL-10 (additional files 3 and 4). NK cells have long been known to be essential for the control of acute *T. gondii* infection [46], but their role in chronic infection maintenance has yet to be elucidated. NK cells are a significant source of IFN-γ during acute *T. gondii* infection [47, 48] and IFN-γ is also necessary to maintain chronic infection [49], but producers of IFN-γ during chronic infection have not been determined. In mice with an established chronic *T. gondii* infection, NK cells are a major source of IFN-γ essential to combat infection with H5N1 influenza virus [50]. Chronic *T. gondii* infection was equally effective to protect against lethal influenza virus whether the mice had been infected with *T. gondii* for 1 month or 4 months, suggesting that NK cells are active in late stages of chronic infection. Similarly, NK are elicited in peritoneal exudate 6 months after *T. gondii* infection [51]. Our data suggests that NK cells play a role in chronic *T. gondii* infection maintenance, which will be the focus of future studies in understanding infection persistence.

Production of nitric oxide (NO) is crucial for control of *T. gondii* growth [52, 53], and triggers differentiation of tachyzoites into bradyzoites in tissue culture [54]. Mice deficient in the inducible nitric oxide synthetase gene succumb to non-lethal doses of *T. gondii,* but only during chronic infection [52, 53]. One host gene differentially expressed during chronic infection is arginase-1 (ARG-1), which had a fold change of nearly 70 in chronic vs. acute samples (Table 6). Arginine is not only a substrate for NO production, but it is an essential amino acid for *T. gondii* [55]. ARG-1 depletes host cell arginine, possibly as means to starve the parasite, but in type I strains, *T. gondii* initiates expression of ARG-1 via ROP16, potentially to preserve infected tissue [31]. While NO is detrimental for parasite growth, it also results in inflammation and subsequent destruction of host tissue. In the brain, microglial cells are the main producers of NO and have the potential to cause neuronal degradation. *T. gondii-*infected astrocytes secrete factors that decrease NO production by microglial cells, thus preserving both host and pathogen during latent infection [56]. It is unclear whether the increase in ARG-1 transcripts is induced by *T. gondii* or the host as ROP16 is more abundant in *T. gondii* during acute infection (Table 3), and ARG-1 is more abundant in the host during chronic infection. Furthermore, ROP16 is polymorphic and in type II strains, such as ME49 used in this study, does not maintain STAT3/6 activation and may not initiate ARG-1 expression [57]. An alternative mechanism may be responsible for the increased abundance of ARG-1 during chronic infection. Another highly differentiated host genes during chronic infection is kallikrein-6 peptidase. Kallikrein peptidases have been implicated in infection through involvement in vasodilation and permeability [58], but in the context of bacterial infections, kallikreins are also involved in the generation of NO [58, 59]. Specifically, kallikrein-6 has been shown to be up-regulated in the CNS during inflammation, possibly as a means to promote lymphocyte survival [60, 61]. Our data suggest kallikrein proteases could be involved in the parasite’s transition from the rapidly replicating form to the encysted form in the host. Together these data suggest that regulation of NO production during chronic infection is of vital importance for both the host and the parasites, and will be a future avenue of research.

## Conclusions

The depth of RNA-seq coverage allowed, for the first time, simultaneous sampling of both host and microbe during acute and chronic stages of animal infection. In this study, we show the majority of highly expressed *T. gondii* genes common to both acute and chronic infection are involved in transcription and translation, underscoring that parasites in both stages are actively synthesizing proteins. Similarly, most of the *T. gondii* genes highly expressed during chronic infection are involved in metabolism, highlighting the metabolic activity of the cyst at 28 days post-infection. For the host, analysis of transcripts at 10 and 28 days post-infection compared to uninfected mice showed that more immunity associated host genes are increased in abundance at 28 days post-infection vs 10 days post-infection. The increase in abundance of *T. gondii* genes during chronic infection is in conjunction with the heightened host response; indicative of the constant battle for survival between the host and the parasite. Discussed here are only a few examples of hypotheses that can be generated from this transcriptome data set. This dataset is novel because information from the host and pathogen is provided at multiple time points, allowing for the interplay between both to be studied. Many platforms, such as KEGG pathways and DAVID, are available for the research community to further investigate these data and cater to their scientific interests. This data provides the potential to elucidate mechanisms required for Apicomplexan parasites to maintain a relationship with their hosts, which will lead to better therapeutics, vaccines and diagnostic methods.

## Methods

### Ethics statement

Animals were housed under conventional, specific-pathogen-free conditions and were treated in compliance with guidelines set by the Institutional Animal Care and Use Committee of the University of Wisconsin School of Medicine and Public Health (IACUC), according to IACUC approved protocol number M01545. This protocol adheres to the regulations and guidelines set by the National Research Council. The University of Wisconsin is accredited by the International Association for Assessment and Accreditation of Laboratory Animal Care.

### IVIS detection of *T. gondii*in the mouse forebrain at 10 days and 28 days post-infection

The ME49 strain of *T. gondii* with a deletion of the gene *HPT* and an insertion of the coding region for firefly luciferase, as previously described [62], was used for these experiments. 6–8 week old BALB/C mice (National Cancer Institute, Charles River Laboratories, Frederick, MD) received an intraperitoneal (i.p.) inoculation of 10^4^ freshly lysed tachyzoites. Mice were imaged using IVIS (PerkinElmer) at 10 days and 28 days post-infection. Mice were anesthetized with isoflurane and intravenous (i.v.) injected with 3 mg of luciferin, the substrate for luciferase, and imaged ventrally, dorsally, and then sacrificed. The brains of the mice were removed and soaked in luciferin for 5 minutes prior to imaging.

### Generation of mRNA and RNA-seq

A ME49 strain of *T. gondii* that was recently passaged through the sexual cycle was used to inoculate mice for RNA-seq analysis. ME49 was maintained as tachyzoites in monolayers of Human Foreskin Fibroblasts in Dulbecco’s Modified Eagle’s Medium supplemented with 10% FBS, 2 mM L-glutamine, and 1% penicillin-streptomycin. 6–8 week old CBA/J mice (National Cancer Institute, Charles River Laboratories, Frederick, MD) were either left uninfected or i.p. injected with 10^4^ parasites and were sacrificed at 10 days and 28 days post-infection. Uninfected mice were sacrificed along with the 28 day post-infection group. We selected *T. gondii* infected mice that were healthy and not displaying any signs of disease, so samples would not contain host transcripts involved with inappetence, dehydration or general malaise to confound our analyses. To minimize changes to the transcriptome, the forebrains were rapidly and precisely sectioned at the intersection of the optic nerves using a mouse brain matrix (Zivic Instruments) with less than one minute between animal sacrifice to forebrain homogenization in 3 mL of TRIzol. Total RNA was isolated according to manufacturer’s protocol. RNA was purified using Promega SV total RNA isolation system according to manufacturer’s protocol. RNA was submitted to the University of Wisconsin Biotechnology Center for purity analysis using the Agilent 2100 Bioanalyzer and sequencing using the Illumina HiSeq2000. Sequencing was performed on each individual mouse and samples were not pooled. Infection was quantified in the un-used hindbrains collected at 28 days post-infection and stained with fluorescein labeled *Dolichos biflorus* agglutinin (Vector Laboratories) for cyst detection. All 28 day post-infection hindbrains contained a minimum of 10,000 cysts.

### Determination of *T. gondii*parasite numbers in mouse forebrain samples

Genomic DNA was extracted from each mouse forebrain at the time of RNA extraction using TRIzol according to manufactures instructions. DNA was purified by phenol/chloroform extraction followed by ethanol precipitation. Genomic DNA was used as the template for quantitative PCR using *T. gondii* primers for the housekeeping gene alpha-tubulin (TUB1A). Tub1A Forward primer 5’-GACGACGCCTTCAACACCTTCTTT-3’, Tub1A Rev 5’-AGTTGTTCGCAGCATCCTCTTTCC-3’. Primer efficiency for TUB1A was 2.002 with an R^2^ value of .99 using *T. gondii* genomic DNA. To determine parasite burden in the mouse forebrain samples, a standard curve was generated using a genomic DNA preparation of known parasite numbers. Quantitative PCR was performed on serial dilutions of parasite genomic DNA, using TUB1A primers, ranging from 10 to 1×10^6^ parasites. A best-fit logarithmic line was generated with an R^2^ of 0.999. The equation of the line along with Ct values obtained from qPCR of TUB1A on genomic DNA from each forebrain sample was used to extrapolate parasite numbers. qPCR was performed on each sample in duplicate using BIO-RAD iTaq Universal SYBR Green Supermix product number 172–5121.

### Quantitative PCR of *T. gondii*CCCH zinc fingers

Sequences for TGME49_224630, TGME49_269270, and TGME49_311100 were obtained from ToxoDB.org. Sequences were run through BLASTp to confirm presence of CCCH zinc finger motifs. cDNA was generated from the same RNA samples used for RNA sequencing with Invitrogen Superscript III Reverse Transcriptase cDNA synthesis kit. All CCCH zinc fingers were normalized to the *T. gondii* house keeping gene tub1A. Efficiencies were determined using in vitro bradyzoite cDNA. RNA was extracted from 5 day bradyzoites grown under low CO_2_ and high pH conditions using TRIzol. cDNA was generated using the Invitrogen Superscript III Reverse Transcriptase cDNA synthesis kit. Efficiencies were calculated using the slopes of a 1:10 dilution series (neat through 10^4^) and the formula *E* = 10^[-1/slope]^. Efficiencies for tub1A, TGME49_224630, TGME49_269270, and TGME49_311100 were 1.96, 1.89, 2.14, and 2.04 (Between 95-107% efficient). Quantitative PCR was performed using Bio-Rad iTaq Universal SYBR Green Supermix on an Applied Biosystems StepOnePlus Real-Time PCR system. Primers were used at a 300 nM concentration and an extension temperature of 60°C for 60 seconds for all primer sets except TGME49_224630 which was run at 56.5°C for 60 seconds. Relative quantification was calculated using Pfaffl’s method [63]. The three biological replicates were used and conducted in duplicate. Wells with only one melt curve and temperature were used, and duplicate Ct values were all at or below a 0.25 difference in cycle threshold value. Primers: Tub1A, same as previously described above

TGME49_224630 Forward 5’-GCGAGGATGAGTGTGGG-3’

Reverse 5’- AGGCGTCACCGTTTGG-3’

TGME49_269270 Forward 5’- GCTTACCGAGGATGACCTGCT-3’

Reverse 5’-CCGTACACTGGTGGCGATCAT-3’

TGME49_311100 Forward 5’- TTTGCCCACACAGCCGAAGAA-3’

Reverse 5’- GCCACAGATGCCTTCCGTAAC-3’.

### RNA-seq, mapping and differential expression analysis

Approximately 950,000,000 paired end 100 bp reads were generated from Illumina HiSeq2000 sequencing. Aligning RNA-seq data for eukaryotic organisms becomes difficult when mapping to a genomic reference because of the presence of introns in the reference and polyadenylated transcripts in the data. If an RNA-seq read spans an exon-exon junction or a polyadenylated region of a transcript it will be "unmappable" to the reference genome and is discarded. Bioinformatics software, such as TopHat, has been created to consider exon-exon boundaries during the mapping process [64]. Raw reads were uploaded onto the Galaxy platform [65–67]. Reads were filtered by Sanger quality score using FASTQ Groomer v. 1.0.4 and paired end reads were aligned against the genomes of *T. gondii* (TGME49 version 9.0; ToxoDB.org) and *M. musculus* (GRCm38 version 74.38; ensemble.org/Mus_musculus) references uploaded into Galaxy using TopHat2 [68]. Parameters for TopHat2: Max edit distance of 2, final read mismatch of 2, anchor length of 8, minimum intron length of 70, maximum intron length of 500000, max insertion and deletion length of 3, number of mismatches allowed of 2, and a minimum length of read segments of 25. Reads were not treated as strand specific as they were paired end reads. The total numbers of reads were as followed: Uninfected mouse 1 was 112075860, Uninfected mouse 2 was 112948998, Uninfected mouse 3 was 103209252, 10 day post-infection mouse 1 was 102581171, 10 day post-infection mouse 2 was 125546828, 10 day post-infection mouse 3 was 81630704, 28 day post-infection mouse 1 was 103765423, 28 day post-infection mouse 2 was 113094439, and 28 day post-infection mouse 3 was 114177191. The number of reads that mapped to the *T. gondii* genomic reference file and *M. musculus* genomic reference files are listed in Table 1.

The program Cufflinks [64] was used to convert aligned reads of BAM files, generated from Tophat2, into relative expression values for each gene represented as FPKM (fragments per kilobase of exon per million mapped reads). Cuffdiff was used to detect significant changes in differential expression between the experimental groups. When running Cuffdiff, a GTF file obtained from ToxoDB.org and ensemble.org/Mus_musculus was used as a guide and the TopHat2 aligned BAM files from each biological replicate were used as the source of comparison between experimental groups. A geometric library normalization and a pooled cross-replicate dispersion estimation method was used when comparing differential expression between each experimental group. Genes with a p-value, for statistical significance, and q-value, to detect the false discovery rate, of <0.05 were considered differentially expressed. The "gene differential testing" output file from Cuffdiff was used to identify differentially expressed genes.

### GO term analysis

FASTA sequences of the most abundant *T. gondii* genes from different time points during infection were loaded into the program Blast2Go [29]. *T. gondii* is not an available organism on many GO term analysis programs, making Blast2Go ideal for uncommon models. Sequences were run with the blastx program against the nr database. Aligned sequences were mapped and assigned GO term annotations. Combined graphs were generated representing the most enriched GO terms in the provided gene list. A score was assigned to determine significance of enrichment. For analysis of *M. musculus,* a gene list was generated from each experimental time point based on gene names in column C of Additional files 2, 3, and 4. Ensembl gene IDs were obtained from these gene names, exported from BioMart and uploaded to the functional annotation tool. A functional annotation chart of the enriched GO terms was generated using GO terms associated with biological process. GO terms were assigned a p-value to indicate significance of enrichment [69]. Only GO terms with a p-value <0.05 were used to represent functional enrichment. For further analysis of *M. musculus,* the online Database for Annotation, Visualization, and Integrated Discovery (DAVID) was used (david.abcc.ncifcrf.gov).

### Availability

Raw short read RNA-seq data has been submitted to ToxoDB and to NCBI with SRA: SRS550800, http://www.ncbi.nlm.nih.gov/biosample/2615816.

## Electronic supplementary material

Additional file 1: **Differential expression analysis of** ***T. gondii*** **genes between acute and chronic time points generated by Cuffdiff.** (XLS 2 MB)

Additional file 2: **Differential expression analysis of** ***M. musculus*** **genes between acute vs. uninfected time points generated by Cuffdiff.** (XLS 16 MB)

Additional file 3: **Differential expression analysis of** ***M. musculus*** **genes between chronic vs. uninfected time points generated by Cuffdiff.** (XLSX 9 MB)

Additional file 4: **Differential expression analysis of** ***M. musculus*** **genes chronic divided by acute time points generated by Cuffdiff.** (XLS 17 MB)

Additional file 5: **Top 5 significant KEGG pathways during chronic vs acute infection.** Genes with a >2 fold abundance between chronic and acute infection were uploaded to the KEGG pathway database. Displayed above are the most 5 most significant pathways enriched during chronic infection. The first column is the KEGG pathway description. Second column is the number of genes that fall into each category. The third column is the p-value designating the significance of each category. (PDF 151 KB)

Additional file 6: **NK cell mediated cytotoxicity KEGG pathway.** Schematic representation of NK cell mediated cytotoxicity provided by Kyoto Encyclopedia of Genes and Genomes ( http://www.genome.jp/kegg/). Red stars indicate genes that are more abundant between chronic and acute infection in the mouse forebrain. For description of features on the map visit http://www.genome.jp/kegg/document/help_pathway.html. (PDF 295 KB)
