# Supplementary material for: Dual transcriptional profiling of mice and Toxoplasma gondii during acute and chronic infection
Source: BMC Genomics. 2014 Sep 20;15(1):806. doi: 10.1186/1471-2164-15-806 (PMC4177681; doi:10.1186/1471-2164-15-806)
Supplement: Supplementary file 5 — Additional file 5: Top 5 significant KEGG pathways during chronic vs acute infection. Genes with a >2 fold abundance between chronic and acute infection were uploaded to the KEGG pathway database. Displayed above are the most 5 most significant pathways enriched during chronic infection. The first column is the KEGG pathway description. Second column is the number of genes that fall into each category. The third column is the p-value designating the significance of each category. (PDF 151 KB) [file 12864_2014_6483_MOESM5_ESM.pdf]

KEGG pathway	number of genes	p-value
cytokine-cytokine receptor interaction	86	6.30E-26
hematopoietic cell lineage	38	1.60E-15
Cell adhesion molecules	53	2.10E-15
graft-versus-host disease	31	3.50E-15
natural killer cell mediated cytotoxicity	45	2.20E-14
