# Supplementary figures and images for: Dual transcriptional profiling of mice and Toxoplasma gondii during acute and chronic infection
Source: BMC Genomics. 2014 Sep 20;15(1):806. doi: 10.1186/1471-2164-15-806 (PMC4177681; doi:10.1186/1471-2164-15-806)

## NATURAL KILLER CELL MEDIATED CYTOTOXICITY

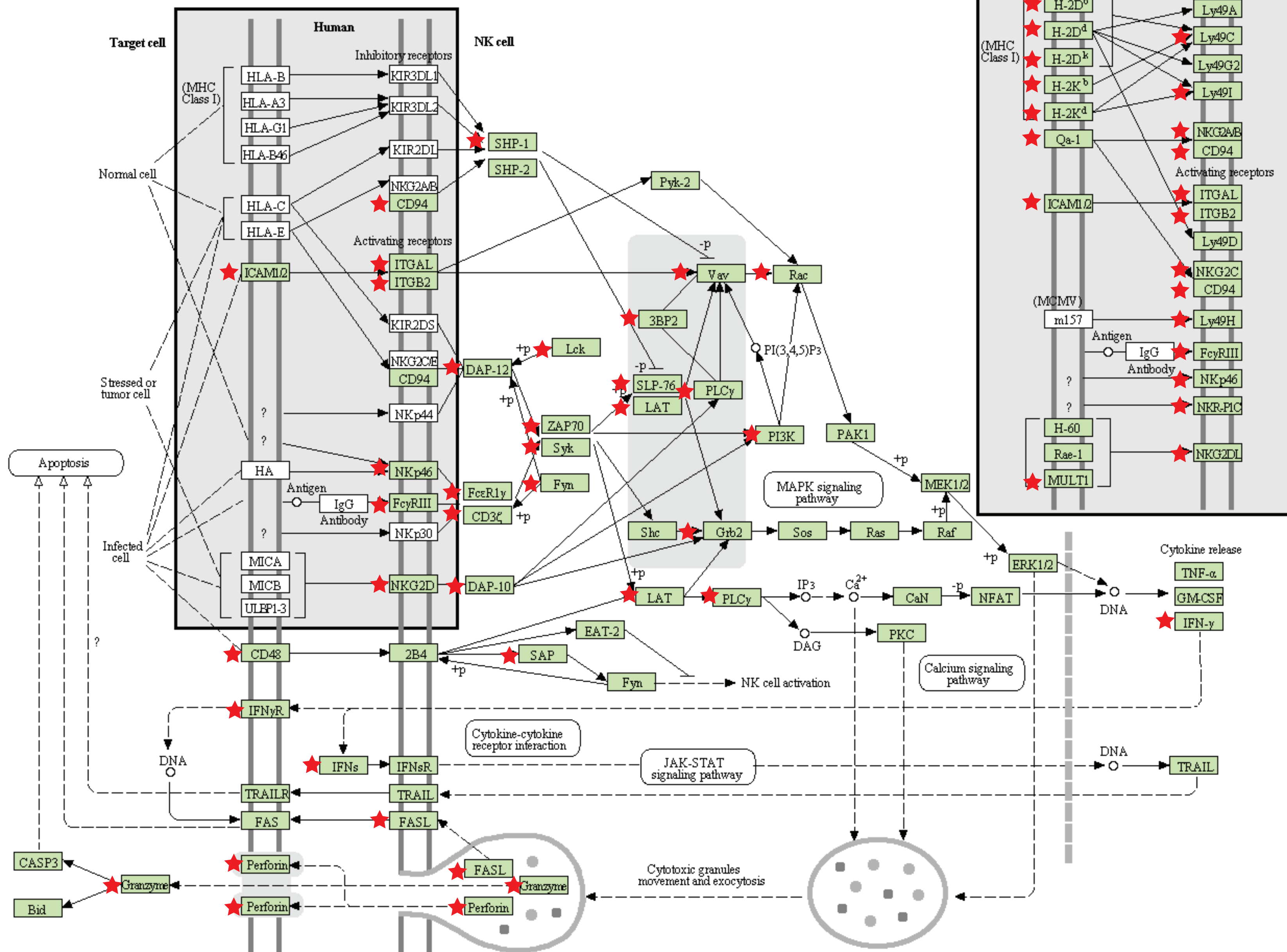

Supplement: Supplementary file 6 — Additional file 6: NK cell mediated cytotoxicity KEGG pathway. Schematic representation of NK cell mediated cytotoxicity provided by Kyoto Encyclopedia of Genes and Genomes ( http://www.genome.jp/kegg/). Red stars indicate genes that are more abundant between chronic and acute infection in the mouse forebrain. For description of features on the map visit http://www.genome.jp/kegg/document/help_pathway.html. (PDF 295 KB) [file 12864_2014_6483_MOESM6_ESM.pdf]
